# Extracellular Vesicles and Their Roles in the Tumor Immune Microenvironment

**DOI:** 10.3390/jcm11236892

**Published:** 2022-11-22

**Authors:** Antonia Reale, Tiffany Khong, Andrew Spencer

**Affiliations:** 1 Myeloma Research Group, Australian Centre for Blood Diseases, Central Clinical School, Monash University—Alfred Health, Melbourne, VIC 3004, Australia; 2 Malignant Haematology and Stem Cell Transplantation, Department of Haematology, Alfred Hospital, Melbourne, VIC 3004, Australia; 3 Department of Clinical Hematology, Monash University, Melbourne, VIC 3004, Australia

**Keywords:** extracellular vesicles, tumor microenvironment, immunosuppression, cancer, immunotherapy, immune-oncology, engineered EVs

## Abstract

Tumor cells actively incorporate molecules (e.g., proteins, lipids, RNA) into particles named extracellular vesicles (EVs). Several groups have demonstrated that EVs can be transferred to target (recipient) cells, making EVs an important means of intercellular communication. Indeed, EVs are able to modulate the functions of target cells by reprogramming signaling pathways. In a cancer context, EVs promote the formation of a supportive tumor microenvironment (TME) and (pre)metastatic niches. Recent studies have revealed that immune cells, tumor cells and their secretome, including EVs, promote changes in the TME and immunosuppressive functions of immune cells (e.g., natural killer, dendritic cells, T and B cells, monocytes, macrophages) that allow tumor cells to establish and propagate. Despite the growing knowledge on EVs and on their roles in cancer and as modulators of the immune response/escape, the translation into clinical practice remains in its early stages, hence requiring improved translational research in the EVs field. Here, we comprehensively review the current knowledge and most recent research on the roles of EVs in tumor immune evasion and immunosuppression in both solid tumors and hematological malignancies. We also highlight the clinical utility of EV-mediated immunosuppression targeting and EV-engineering. Importantly, we discuss the controversial role of EVs in cancer biology, current limitations and future perspectives to further the EV knowledge into clinical practice.

## 1. Introduction

### 1.1. Extracellular Vesicles

Extracellular vesicles (EVs) are particles delimited by a lipid bilayer, typically described as small EVs (e.g., exosomes, ~100 nm in diameter) and medium-large EVs (shed microvesicles, 200 nm to ~1300 nm) [1,2]. EVs are released from all cell types into the extracellular space and play important roles in both physiological and pathological processes including cancer [1,3,4,5,6,7].

Cells actively incorporate factors (e.g., proteins, lipids, RNA) into EVs which can be transferred to target (recipient) cells, making EVs an important means of intercellular communication [8,9,10,11]. Indeed, EVs are able to modulate the function of recipient cells by reprogramming signaling pathways. In cancer, EVs are able to promote the formation of a supportive tumor microenvironment (TME) and (pre)metastatic niches [8,9,12,13,14,15,16]. Importantly, EVs protect their molecular content (cargo) from degradation in the extracellular space and can be collected (isolated, enriched and purified) for downstream applications/analyses from biofluids such as blood and urine, making them ideal candidates for liquid biopsy [7,17,18]. In addition, EVs express common surface markers with their cell of origin, designating them as ideal candidates not only for biomarker discovery and diagnostics but also for therapeutics in cancer [7,8,17]. 

### 1.2. The Tumor Microenvironment

The existence of dynamic interactions of cancer cells with the (tumor) microenvironment is well established. The TME consists of cellular components including fibroblasts, endothelial cells, immune cells (e.g., macrophages, T and B lymphocytes, dendritic cells-DC), non-cellular components including soluble factors (e.g., cytokines, growth factors, chemokines), extracellular matrix (ECM) proteins (laminin, fibronectin and collagen) and EVs. The TME is essential for stimulation of the heterogeneity of cancer cell, clonal evolution and to promote multidrug resistance ultimately favoring cancer progression and metastasis formation. “In the specialized TME, cells are protected from apoptotic stimuli and may, therefore, actively promote cancer progression as well as treatment failure” [19,20,21,22,23,24].

### 1.3. EVs and the Tumor Immune Microenvironment

Accumulating evidence has confirmed immune evasion to be one of the hallmarks of tumor progression both in solid and blood cancers [25,26]. Recent studies have established immunosuppressive activities of immune cells that allow tumor cells to establish, proliferate and disseminate [27]. Tumor cells are capable of evading immune cells by releasing immunosuppressive molecules (e.g., interleukin IL-10, transforming growth factor β—TGF-β) or by loss of adhesion molecules such as ICAM-1. Tumor cells are able to develop resistance to apoptosis by overexpressing BCL-2 and other anti-apoptosis molecules, or immune inhibitory receptor ligands such as programmed death ligand 1 (PD-L1), Fas ligand and tumor necrosis factor (TNF)-related apoptosis-inducing ligand (TRAIL) [28]. Furthermore, tumor antigens are heterogeneous and have high mutation rates making immune cells unable to recognize and kill tumor cells. Cancer immunotherapy, by enhancing the immune system function in cancer patients to effectively kill tumor cells, has recently emerged as a novel successful therapeutic strategy [29,30,31,32,33]. 

The ability of cancer cells to use EVs to establish an ideal TME for disease progression [34,35,36,37,38,39,40] has generated particular interest. We have recently demonstrated, in our model of multiple myeloma, a hematological malignancy, that in response to their interaction with tumor-derived EVs (TEVs), human naïve stromal cells acquire pro-proliferative and pro-migratory phenotypes, with subsequent increase in tumor cells’ adhesion to pre-treated stromal cells [18]. We also demonstrated, for the first time in myeloma, that the depletion of albumin in EVs derived from blood plasma can improve protein detection by mass spectrometry. Importantly, by using this optimized proteomic approach, we demonstrated that TEVs collected from plasma of patients with myeloma are enriched in proteins associated with cell migration and cell adhesion among other biological processes [6,18]. Unpublished data indicate that TEVs collected from plasma of patients with myeloma are also enriched in factors which may induce immune suppression and tumor-promoting inflammation. This is consistent with a recent work by Lyden et al. [7] describing the proteome profile of TEVs derived from solid cancers. The authors describe molecules specific to TEVs, such as S100A4, S100A13, BSG, LGALS9, which may induce immune suppression and tumor-promoting inflammation. Lyden et al. [8,9] have previously demonstrated that TEVs are transferred to recipient cells at distant sites and by releasing their content TEVs can generate pro-inflammatory (pre)metastatic niches that support future metastasis.

TEVs have been shown to induce immunosuppression in several other cancer models [41,42,43,44,45]. Buzas [12] has recently provided a broad overview on the roles of EVs in physio-pathological mechanisms of innate and adaptive immunity, including inflammation, antigen presentation and the development and activation of B cells and T cells. The author highlights important progress related to several pathological conditions (e.g., in antimicrobial defence and in allergic, autoimmune responses) with a brief summary on the role of EVs in anti-tumor immune responses.

In this review, we discuss the current knowledge derived from analyses of these particles and their roles in tumor immunosuppression. We also discuss the clinical utility of EVs in immunotherapy and the limitations of their applicability in clinical settings. Methodological difficulties and inconsistencies in rigor and reporting have hampered the translation of research findings into clinical practice. Recently, significant progress has been made in techniques for the isolation, enrichment and characterization of EVs, but further improvement is required, particularly when attributing specific functions to EVs versus other factors present in the extracellular space that are often co-isolated. Data described in this review confirm the role of EVs as key modulators of immune functions in the TME, hence a better understanding of cellular and molecular mechanisms governing the EV-mediated interactions may provide important insights for developing new strategies to disrupt the cell-to-cell communication within the TME and inform new therapeutic strategies to fight solid and blood cancers.

## 2. EVs and Tumor Immune Suppression

Recent exiting observations have suggested that EVs derived from tumor cells express PDL1, a natural ligand for programmed death 1 (PD1) [46]. Tumor-derived PDL1 can suppress the functions of immune cells by directly binding with PD1 on their surface, hence promoting tumor immune escape [47,48,49]. Blocking the interaction between PD1 and PDL1 has been of revolutionary significance in cancer therapy, resulting in approvals for PD1/PDL1 blocking agents from the US Food and Drug Administration (FDA) for the treatment of several cancers [50]. A recent review [51] has extensively described the role of PDL1 in EV-mediated tumor immune evasion. As PDL1-positive EVs can impair immune activities and support tumor growth in a similar way as tumor-derived PDL1, targeting EV-PDL1 may augment the anti-tumor memory response and overcome resistance to PD1 blockade. In addition, EV-PDL1 has been shown to serve as early indicator of clinical benefit in melanoma [52]. As biomarker, EV-PDL1 may allow for rational implementation of therapies to minimize toxicity and maximize clinical benefit.

**Figure 1:**
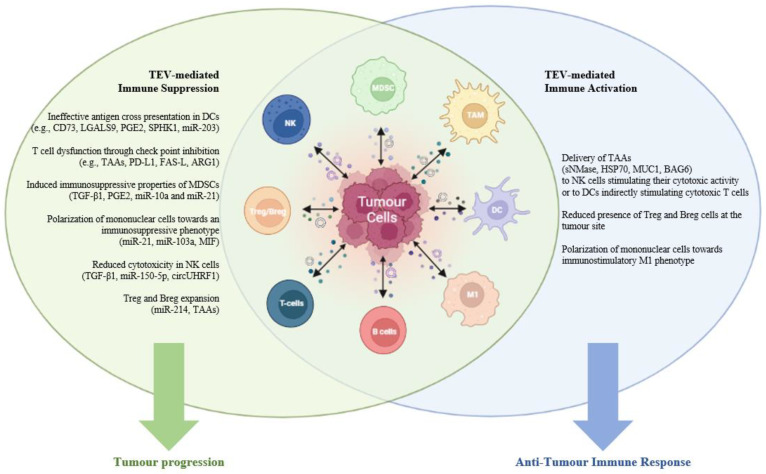
Tumor-derived EVs regulate immune system cells’ functions. TEVs can promote tumor progression by suppression of innate and adaptive immune cells, as indicated in the left green panel. In fact, TEVs hamper effective antigen cross presentation in DCs, contribute to T cell dysfunction through check point inhibition, polarize mononuclear cells towards an immunosuppressive phenotype, induce immunosuppressive properties of MDSCs, promote Treg/Breg expansion and reduce NK cells cytotoxicity activity. TEVs can also promote tumor recognition enhancing anti-tumor immune functions, as shown in the right blue panel. During a functional immune response, TEVs deliver TAAs to NK cells to stimulate their cytotoxic function or to DCs to indirectly stimulate cytotoxic T cells, to polarize mononuclear cells towards an immunosuppressive phenotype. Macrophages can actively uptake TEVs from circulation promoting cancer cell recognition. Examples of molecules involved in these mechanisms are also indicated. (EVs, tumor-derived extracellular vesicles; TAAs, tumor-associated antigens; NK, natural killer; Treg, regulatory T cells; MDSC, myeloid-derived suppressor cells; DCs, dendritic cells; sNMase, neutral sphingomyelinase; HSP70, heat shock protein 70; MUC1, mucin-1; BAG6, large proline-rich protein BAG6; PD-L1, programmed death-ligand 1; FAS-L, tumor necrosis factor ligand superfamily, member 6; ARG1, arginase-1; TGF-β, transforming growth factor beta; LGALS9, galectin 9; MIF, macrophage migration inhibitory factor; SPHK1, sphingosine kinase 1, PGE2, prostaglandin E2). Figure generated with the aid of BioRender.com (adapted from “Pro- and Anti-Tumor Immune Cells in the Tumor Microenvironment biorender”, accessed on 15 November 2022).

In this section, we will describe TEV interactions with immune cells which activate downstream signaling resulting in molecular and functional alterations of the recipient cell. These interactions have a profound impact on cancer development, progression, metastasis and emergence of drug resistance. Delineating the cell types, molecules and functions present in the TME will support the development of more effective treatments. A summary of these mechanisms can be found in Table 1 and Figure 1.

### 2.1. EVs and Tumor Immune Suppression: Role of T and B Cells

As recently described in a review by Vergani et al. [43] “tumor cells are able to escape recognition by T cells by modifying their immunogenic profile, for example by antigen loss or accumulation of mutations in genes involved in immune recognition” [118]. Tumor cells can acquire the expression of molecules with ‘don’t eat me’ function (i.e., CD47), leading to inhibition of their phagocytosis by macrophages [119]. Tumor cells can acquire immunosuppressive factors to directly kill T cells (i.e., FasL, TRAIL) and can overexpress immune checkpoint molecules (ICP), e.g., PD-L1, thereby affecting the functions of activated CD8^+^ T cells and promoting T cell exhaustion [120]. Immune suppressive molecules enriched in TEVs can be released into the circulation exerting immune suppressive functions also at distant (metastatic) sites from the primary tumor site [43]. In a study by Dou X et al. [53], small cell lung cancer (SCLC) cells expressing high levels of PD-L1 released TEVs overexpressing PD-L1. The latter induced downregulation of the T-cell activation marker CD69 on CD8^+^ T cells and also modulated the release of various cytokines from stimulated cells. Importantly, the inhibition of CD8^+^ T cell activation mediated by EVs was significantly reversed by an anti-PDL1 blocking antibody. The authors also performed a retrospective study showing that TEV-PDL1 was an independent prognostic factor and significantly correlated with progression-free survival in SCLC patients. These results corroborate findings by Theodoraki et al. [121], where EV-PDL1 can serve as a diagnostic biomarker for predicting the effectiveness of therapy, as well as a new strategy to enhance T-cell-mediated immunotherapy against SCLC cancers. TEV-PDL1 has also been described in melanoma, glioblastoma, prostate cancer, NSCLC, head and neck cancers, breast and gastric cancers [47,59,60,61,62,64].

In addition to T cells suppression mediated by EV-PDL1, other mechanisms have been described [65,67,68,69,70,72]. For instance, Czystowska-Kuzmicz et al. [48] have confirmed that ovarian cancer cells use TEVs to deliver the metabolic checkpoint factor ARG1 to DCs in LNs, hence inhibiting antigen-specific T cell proliferation. The authors observed that overexpression of ARG1 in mouse ovarian cancer cells is associated with accelerated tumor progression and that the latter can be inhibited by an arginase inhibitor. Gupta P et al. [54] describe TEVs as contributors of ovarian cancer progression mediated by sphingosine kinase-1 (SPHK1). In this previously unrecognized mechanism, TEV-SPHK1 upregulate S1P levels in the TME, where S1P induces T cell exhaustion and is also able to upregulate PD-L1 expression through E2F1-mediated transcription. Importantly, the SPHK1 inhibitor PF543 improved T cell-mediated cytotoxicity. The authors also show positive effects when combining PF543 with an anti-PD-1 antibody on tumor burden and metastasis vs. PF543 alone in vivo. Hence, improved immune checkpoint inhibition by targeting SPHK1/S1P signaling has potential clinical application in ovarian cancer. Xie F et al. [55] observed that breast cancer cells secrete TEV enriched in active TGF-β type II receptor (TβRII) which transfer to CD8^+^ T cells induced the activation of transforming growth factor-beta signaling protein 3 (SMAD3). SMAD3 cooperates with transcription factor TCF1 to promote CD8^+^ T cell exhaustion. This results in failure of immunotherapy. These findings identify a new possible targetable mechanism by which breast cancer cells induce TEV-mediated T cell exhaustion and negatively affect anti-tumor immunity.

Vignard et al. [56] found that miRNAs enriched in melanoma-EVs (e.g., miR-122, miR149, miR-3187-3p, miR-181a/b, miR-498) downregulate T-cell responses and cytotoxic activity by decreasing T-cell receptor (TCR) signaling and granzyme B and cytokine secretions. These observations suggest that miRNAs in melanoma-derived EVs promote tumor immune evasion and may represent a therapeutic target. Leukemic cells derived from patients with acute myeloid leukemia (AML) carrying nucleo-phosmin (NPM1) secrete EVs enriched in miR-19a-3p. The latter targets SLC6A8-mediated creatine import and promotes immunosuppressive activities of CD8^+^ T cells. These observations indicate that TEV-miR-19a-3p might be a promising therapeutic target for AML carrying NPM1 mutations [57].

Both the significance of humoral immune responses and the processes through which tumor associated antigens (TAAs) are recognized by the immune system and trigger a humoral response remain undefined to date. As evidenced by the production of auto-antibodies against TAAs, B cell-associated auto-immune responses are found in several tumor types. It is suggested that tumor cells express certain TAAs in their TEVs in order to divert the humoral immune response away from the tumor, thereby preventing its effective detection and elimination [74,122]. Capello et al. [74] observed that pancreatic ductal adenocarcinoma (PDAC)-EVs expose several TAAs and bind auto-antibodies present in PDAC patient blood, exerting a broad decoy-like function. Hence, complement-dependent cytotoxicity and potentially antibody-dependent cell-mediated cytotoxicity are inhibited. Similarly, it has been shown that “TEV mediate the sequestration of anti-CD20 [123] and anti-Her2 [124] therapeutic antibodies impairing antibody-based cytotoxicity directed at cancer cells and promoting resistance to cell surface directed therapeutic antibodies. These observations might explain the limited success of EV-based cancer immunotherapies and provide new insights into TEV-mediated immune escape processes with further investigation warranted”.

### 2.2. EVs and Tumor Immune Suppression: Role of Tregs

Tumor sites and draining LNs are also embedded with regulatory T cells (Tregs) [125], specialized immune suppressive cellular subsets, which compete with antigen specific CD8^+^ T cells [125,126]. Increased Treg/Tconv (conventional T cells) and Treg/CD8^+^ T cell ratios at the tumor site have often been described and are associated with poor prognosis in several cancers [127]. As the depletion of Treg cells significantly reduces tumor burden [128,129,130], strategies targeting receptors (CCR4, CD25, CTLA-4, CCR8) preferentially expressed on tumor infiltrating Tregs [125,128,131] are currently being evaluated in clinical trials.

TEV have been shown to induce the suppression of anti-tumor immune responses by also modulating the actions of Treg [58,70,75,116,132,133,134]. Indeed, TEV containing TGF-β1 induce Treg cells [134]. Ning et al. [135] demonstrated that TEVs derived from colorectal cancer (CRC) cells containing miR-208b promoted Treg expansion by targeting programmed cell death factor 4 (PDCD4). Treg expansion mediated by TEV-miR-208b resulted in tumor growth and oxaliplatin resistance. These findings highlight a potential role of TEV-miR-208b as a novel target for immunotherapy. CD73 + γδT1 cells have been found to be the predominant Treg population in breast cancer [71], with their prevalence in circulation found to be related to tumor burden. Breast cancer-derived TEV could transmit long non-coding (lnc)RNA SNHG16 to Vδ1 T cells. SNHG16 served as a competing endogenous RNA by sponging miR-16–5p, which led to the de-repression of its target gene SMAD5 and resulted in potentiation of the TGF-β1/SMAD5 pathway to upregulate CD73 expression in Vδ1 T cells. These observations clarify the significance of CD73 + Vδ1 Tregs in breast cancer and identify a new potential target (Treg subpopulation or TEV) with potential benefit for patients with breast cancer. TEV isolated from supernatants of head and neck squamous (HNSCC) cancer cells and ovarian cancer cells but not normal cells induced the generation and enhanced expansion of human Treg [136]. When Treg were co-incubated with TEVs, overexpression of FasL, IL-10, TGF-β1, CTLA-4, granzyme B and perforin was observed as well as inhibition of T cell proliferation. Purified Treg were resistant to TEV-mediated apoptosis relative to other T cells. The ability of TEV to promote Treg expansion was counteracted by neutralizing antibodies specific for TGF-β1 and/or IL-10. In line with these observations, other researchers have shown that EVs from the plasma of patients with advanced stages of HNSCCs exhibit immune suppression functions through the synthesis of adenosine, expansion of Treg and induction of immunosuppressive phenotype of CD8^+^ T cells mediated by galectin-1 [59,137] and also by inducing apoptosis of CD8^+^ T cells and suppression of CD4^+^ T cell proliferation [46]. These EVs were shown to carry immunosuppressive proteins, including PD-1, PD-L1, Fas, FasL, CTLA-4, TRAIL, CD73, COX2, TGFβ-LAP [46]. These data can explain the high speed of HNSCC cancer formation and its high rate of cancer recurrence and inform TEVs as potential therapeutic targets to prevent the dysfunction of T cells and promote anti-tumor immune responses.

TEV from nasopharyngeal carcinoma were shown to transfer miRNA (miR-24-3p; miR-891a; miR-106a-5p; miR-20a-5p) to promote downregulation of ERK/STAT1/STAT3 phosphorylation with a shift of T cells towards Treg phenotypes [138].

TEV interacting with immune cells create an immunoinhibitory microenvironment favoring immune escape also in blood cancers. Chronic and acute myeloid leukemia evade immune system surveillance and induce immunosuppression by expanding pro-leukemic Foxp3+ Tregs. High levels of immunosuppressive Tregs predict inferior response to chemotherapy, leukemia relapse and shorter survival [139]. In a recent study Swatler et al. [140] identified leukemic TEVs as drivers of effector pro-leukemic Tregs which expressed CD39, CCR8, CD30, TNFR2, CCR4, TIGIT, IL21R. Furthermore, the authors observed that leukemic EVs shuttled co-stimulatory ligand 4-1BBL/CD137L regulating expression of receptors such as CD30 and TNFR2 and ultimately promoting suppressive functions and effector phenotype of Tregs. The authors describe a Rab27a-dependent secretion of EVs and suggest that targeting of Rab27a-dependent secretion of leukemic EVs may be a viable therapeutic approach in myeloid leukemia. 

### 2.3. EVs and Tumor Immune Suppression: Role of Natural Killer Cells

As described in a review by Carlsten and Järås [141] natural killer (NK) cells, large granular lymphocytes, are involved in immune defense against cells infected by viruses and tumor cells. The anti-tumor actions of NK cells are based on signals from activating and inhibitory cell surface receptors. NK cells are also able to lyse target cells via antibody-dependent cellular cytotoxicity which represents a critical mode of action of several therapeutic anti-cancer antibodies [142,143]. Cancer-associated mechanisms often restrain the proper activities of NK cells, leading to inadequate disease control and cancer progression. NKG2D, the classic activating NK receptor, render tumor cells susceptible to NK cell-mediated cytolysis when expressed at high levels. However, cancer cells are able to evade NKG2D-mediated immunosurveillance by shedding NKG2D ligand (NKG2DL), hence with reduced expression levels of NKG2D [144].

As shown by several groups [145,146,147], soluble NKG2DL (e.g., MICA, MICB, ULBP1-2) and TEV expressing NKG2D-L trigger the reduction of NK cell surface NKG2D.

Whiteside et al. [148] have demonstrated for the first time in 2011 inhibition of NK cell-mediated lysis of leukemic K-562 cells by TEVs isolated from patient serum. They further demonstrated that circulating TEVs from AML patients targeted purified healthy donor-derived NK cells directly resulting in down-regulation of NKG2D. TEV were shown to carry TGF-β1 which antibody-mediated neutralization inhibited suppressive activities of AML-TEVs, confirming that TEV-TGF-β1 promoted NK-cell dysfunction [149,150,151]. These observations suggest that persistently elevated circulating levels of biologically functional AML-TEVs impair immune responses and contribute to leukemia progression. AML-TEVs were also shown to bind to therapeutic NK-92 cells with activation of intracellular signaling leading to a lack of therapeutic efficiency during adoptive cell therapy [149]. Furthermore, chemotherapy significantly increase the secretion of AML-TEVs, thereby potentially contributing to therapy resistance as confirmed by other groups [152,153].

TEVs carrying miR-23a have been shown to reduce expression of lysosomal-associated membrane protein 1 (CD107a/LAMP1), an NK cell activation marker, in leukemia and lung adenocarcinoma. TGF-β and miR-23a enriched in hypoxia-induced TEV induce NK cell suppression by downregulating CD107a and NKG2D in vitro [154,155]. The immunosuppressive role of EVs is also described in solid tumors such as PDAC, lung cancer and hepatocellular carcinoma [81,82,156], with a better understanding of metastasis formation, cancer progression and drug resistance mediated by NK cell dysfunction.

### 2.4. EVs and Tumor Immune Suppression: Role of MDSCs

Myeloid-derived suppressor cells (MDSCs) are a heterogeneous population of immature myeloid cells with suppressive functions, containing myeloid progenitor cells and granulocyte precursors, macrophages and DCs. Elevated MDSCs levels in the blood of cancer patients are associated with inhibited T cell proliferation and reduced IFNγ production. The proportion of MDSCs in blood correlates with tumor burden and is inversely associated with immune checkpoint inhibitors (ICIs) response [90,91,116,132,133,157,158]. TEV have been shown to activate MDSCs [79] and to upregulate PD-L1, Cox2, IL-6, VEGF, arginase-1, NF-kB, TLR4 expression on MDSCs [90,91]. Furthermore, TEV-miRNA are able to alter myeloid cells to myeloid-derived suppressor cells or tumor-associated M2 macrophages (TAMs) and promote the malignant behaviour of cancers [76,77,78,80,89,159]. Melanoma-derived EVs, enriched in HSP86, induced the generation of PD-L1 + CD11b + Gr1 + MDSCs that suppressed T cell functions [90]. Another recent work suggests the presence of hepatoma-derived growth factor (HDGF) in the culture medium of human multiple myeloma (MM) cell lines, cell lysates and TEVs. The authors demonstrated that HDGF acts in an autocrine fashion activating the AKT pathway in MM cells, hence maintaining proliferation and playing a significant role in metabolism. The authors also describe a potential paracrine role where HDGF affects cells in the TME including macrophages and immature monocytes. HDGF induces macrophages’ polarization to an M1-like phenotype and alters naïve CD14^+^ monocytes to functionally suppressive MDSCs [116].

### 2.5. EVs and Tumor Immune Suppression: Role of Antigen Presenting Cells

DCs, the major type of antigen presenting cells (APCs), are involved in both innate and adaptive immunity. Their main function is to process antigens and present them to T cells initiating the adaptive immune response. Immature DCs (iDC) express high levels of chemokine receptors (CCRs) and low levels of co-stimulatory factors, capture and process antigens and migrate to LNs where they acquire antigen presenting capabilities. Here, DCs secrete cytokines and express high levels of co-stimulatory factors (e.g., CD80, CD86). This differentiation process is critical for DCs to become APCs and activate adaptive immune responses. In cancer pathophysiology, DCs orchestrate the immune reactions in the tumor and TEVs have been shown to promote immune dysfunction in DCs both in solid and blood cancer models [88,160,161].

Binding of galectin 9 (LGALS9) expressed on EVs derived from glioblastoma multiforme (GBM) cells to the TIM3 receptor of DCs affects antigen recognition, processing and presentation, leading to inhibition of cytotoxic T cell-mediated anti-tumor responses [85]. In a recent study, de Paula Silva et al. [86] observed significant effects on DCs function when DCs were treated with TEVs enriched from squamous cell carcinoma (SCC). The authors describe reduced inflammatory response and expression of lipopolysaccharide (LPS) response in addition to reduced chemokine and chemokine-mediated signaling and cytokine-cytokine receptor interaction. The authors identified EV-miRNAs as mediators of DC dysfunction (by targeting genes such as tumor necrosis factor-α—TNF-α, IL-12, IL-14), including inhibition of co-stimulatory molecules, antigen processing and presentation, DC maturation, migration, reduced production of inflammatory cytokines and increased DC death. In PDAC, EVs carrying miR-203 promote tumorigenesis by inhibition of the functions of DCs. EV-miR203 downregulates Toll-like receptor 4 (TLR4), IL-12 and TNF-α expression, hence preventing antigen presentation by DCs [84]. Similarly, EVs enriched from pancreatic cancer cells contain miR-212-3p which, transferred by DCs, downregulates the expression of regulatory factor X-associated protein (RFXAP). The latter decreases MHC class II expression with associated inactivation of CD4^+^ T cells, contributing to the generation of an immunotolerant TME in PDAC [83]. Targeting EV-miRNAs may represent a new strategy to improve treatment outcomes in patients with cancer.

Salimu et al. [87] have demonstrated that EVs containing the lipid mediator prostaglandin E2 (PGE2) derived from prostate cancer cells induced DCs to express the immunosuppressive marker CD73 on their surface. This significantly impacts on cytokine production and T cell activation. DCs expressing both CD73 and CD39 are able to hydrolyze ATP to adenosine in the TME. Adenosine has direct tumor-promoting, angiogenic and metastasis-inducing effects and is also a powerful inhibitor of anti-tumor immune effector cells in the TME. It also impairs maturation and function of DCs. “Although TEV have been reported to generate anti-tumor immune responses in several murine tumor models, the results from Salimu et al. strongly support the notion that, even if antigen is delivered by TEV to DC, TEV immunosuppressive properties override the potential antigen-delivery function. Indeed, a clinical trial with TEV as a cancer vaccine in patients with colorectal cancer [162] found no clinical benefit, reflecting the immunosuppressive effects of TEV”.

### 2.6. EVs and Tumor Immune Suppression: Role of Monocytes and Macrophages

Monocytes, an innate immune cell population, are able to either suppress or promote anti-tumor immunity depending on the context. Monocytes can differentiate into TAMs and DCs, promote angiogenesis, remodel the extracellular matrix, kill tumor cells and recruit lymphocytes.

Macrophages are the most abundant immune cells in TME and can polarize from M1 to M2 cells (TAMs) depending on physiological or pathological conditions. “While M1 macrophages (induced by T helper type 1-like cytokines such as IFN-γ) produce pro-inflammatory cytokines, chemokines and reactive nitrogen/oxygen intermediates and are involved in antimicrobial and tumoricidal activity, M2 TAMs (induced by IL-4 and IL-13) show anti-inflammatory and tumor-promoting activities. TAM represent the majority of macrophages present in the TME and a high number of TAM in various solid tumors is associated with poor prognosis and metastasis formation” [163,164].

The transition from conventional pro-inflammatory M1 to immunosuppressive M2 TAMs is a highly dynamic process regulated by several cellular signaling pathways in the TME. Accumulating evidence has established that TEVs are involved in this dynamic polarization form M1 to a M2 phenotype in cancer [80,96,98,101,103,105,108,109,164]. For instance, RNA sequencing of TEVs derived from chronic lymphocytic leukemia (CLL) revealed a high enrichment of non-coding Y RNA hY4 which transfer increased the release of cytokines [e.g., C-C motif chemokine ligand 2 (CCL2), CCL4, IL-6] and the expression of PDL1 expression in circulating monocytes via TLR7 signaling [49]. Pharmacologic inhibition of endosomal TLR signaling resulted in a substantially reduced activation of monocytes in vitro and attenuated CLL development in vivo.

Huber et al. [89] reported that melanoma-derived EVs enriched in miRNA (miR-155, miR-125b, miR-100, miR-146a, miR-146b, let-7e, miR-125a and miR-99b) conferred immunosuppressive properties to monocytes derived from healthy donors. The authors also observed that baseline levels of circulating EV-miRNA were predictive of ICIs resistance. TEVs from glioblastoma-derived stem cells (GSC) have also been shown to induce an immunosuppressive phenotype by promoting monocyte differentiation into M2 TAMs and enhancing the levels of PD-L1 [114]. Similarly, TEVs from gastric cancer-mediated monocyte differentiation into PD1 + TAMs with M2 properties in vitro and in vivo [115]. Notably, PD1 + TAM correlated with poor prognosis in gastric cancer and suppressed CD8^+^ T cell functions. In pancreatic cancer patients, immunosuppressive circulating CD14 + HLA-DRlo/neg monocytes were increased when compared to healthy controls. Importantly, TEV mediated the down-regulation of HLA-DR in monocytes and induced arginase expression and ROS [112]. The overexpression of the EMT transcriptional factor Snail in HNSCC cells enhanced the production of TEV-miR-21 which, transferred to monocytes, induced the downregulation of M1 markers and the increase of M2 markers [113]. Knockdown of miR-21 in HNSCC cells decreased tumor growth, M2 infiltration and angiogenesis in an in vivo model. Interestingly, a high expression of miR-21 correlated with increased SNAI1 and with M2 polarization in HNSCC patient samples. 

Popēna et al. [104] have shown that macrophages exposed to colorectal cancer cell-derived TEVs displayed increased CXCL10 secretion and levels of the surface marker CD14, while monocytes displayed increased CXCL10, TNF-α and IL-1β secretion. Noteworthy, monocytes from patients with advanced cancer secreted significantly more TNFα than monocytes from patients at an early stage of the disease. Furthermore, an increase in the frequency of CD14 + CD169 + macrophages is associated with the development and progression of colorectal cancer and a higher level of both macrophages’ phenotypes (TAMs and M1). Elevated levels of CXCL10 in the serum of patients with colorectal cancer correlated with liver metastasis and poor survival. In this model TEV promote the polarization of inactive (M0) macrophages to both M1 and TAM with secretion of CXCL10, IL-6, IL-23 (M1) or IL-10 (TAM), supporting the notion that increased levels of circulating CXCL10 and IL-6 are associated with advanced stage of disease and that IL-6 is an independent prognostic marker of poor survival. The authors have also analysed the internalization of TEV by monocytes and macrophages and suggest that the endocytosis of TEVs occurs via phagocytosis and endocytic pathways dependent on dynamin. By elucidating such mechanisms, new therapeutic avenues may be informed.

Lung cancer-derived TEVs can deliver miR-21 and miR-29a to macrophage activating TLR8 and the NFKB pathway and leading to the secretion of IL6 and TFNA with associated pro-tumor inflammatory environment [110]. Similarly, miR-21 from neuroblastoma-TEV could again trigger TLR8 in monocytes, which led to upregulation of miR-155 in those cells. miR-155 could then be passaged back to the tumor cells via EVs downregulating the miR-155 target telomeric repeat binding factor 1 (TERF1), a telomerase inhibitor, with subsequent upregulation of telomerase which promotes neuroblastoma drug resistance [117]. Gerloff et al. [97] showed for the first time that miR-125b-5p transferred by cutaneous melanoma-derived EVs induces a tumor-promoting TAM phenotype in macrophages.

Recently, Wang & Gao [100] have shown that pancreatic cancer cell-derived EVs transferred miR-155-5p to macrophages and then promoted polarization to TAM phenotype. The authors describe a previously unrecognized tumor immune evasion-promoting function of TEV-miR-155-5p which suppressed EHF resulting in activation of the Akt/NF-κB signaling. This study suggested that the miR-155-5p/EHF/Akt/NF-κB axis can be exploited to prevent cancer immune evasion triggered by therapies. 

Hypoxia, an intrinsic property of several cancers, induces increased secretion of EVs often enriched in immunosuppressive proteins and miRNA. Indeed, TEV-mediated polarization of macrophages to TAMs in a PDL1/HIF-1α-dependent manner in a non-small-cell lung cancer model was shown [102]. Hypoxic TEVs isolated from melanoma cells promoted TAM polarization in infiltrating macrophages in a syngeneic mouse model of macrophage infiltration [95]. In addition, miR-103a and Let-7a, enriched in TEVs at higher levels under hypoxic conditions, have been shown to promote TAM polarization in lung, skin and melanoma cancers [93,95]. Epithelial ovarian cancer (EOC) cells secrete TEVs that are able to promote macrophage polarization under hypoxic conditions by transferring miRNAs (miR-21-3p, miR-125b-5p, miR-181d-5p), hence promoting tumor proliferation and metastasis [111]. 

Moreover, there is evidence that TEVs transfer growth factor receptors to leukocytes impairing the antiviral immunity of patients with cancer. Lung cancer-derived TEVs can transfer activated EGFR molecules to macrophages activating MEK kinase 2 (MEKK2), which negatively regulates the antiviral immune response. This mechanism may explain the immunocompromised status of cancer patients [94].

## 3. Immune Cells-Derived EVs

EVs produced by immune cells can participate in tumor immune responses [44]. EVs derived by T cells have been shown to be involved in invasion and metastasis processes of several cancers. For example, EVs isolated from activated CD8^+^ T cells express Fas and promote melanoma and lung cancer cells invasion via the Fas/FasL pathway [165].

Plasma of patients with HNSCC contained a high number of CD3^+^ T cell-derived EVs vs. healthy donors. These EVs showed high expression of CD15s, expressed by Treg, suggesting that the EVs increase may be correlated to the expansion of this immune suppressive cell subtype [166].

In a recent work, Chiou et al. [167] have shown that EVs derived from activated T cells are enriched in small RNA fragments (tRNA, tRF) that inhibit the activation of T cells. Hence, T cells may selectively secrete EV-tRF that can inhibit their activation. EVs enriched in tRFs may be found in circulation and may represent biomarkers of ongoing immune activation during anti-tumor immune responses.

NK EVs have been shown to be enriched in the tumor suppressor miR-186, which was downregulated in MYCN-amplified neuroblastoma cell lines and in tumor lesions from high-risk patients vs. low-risk patients [168]. Fenselau & Ostrand-Rosenberg [169] observed that MDSC-EVs expressed molecules such as S100A8 and S100A9 and displayed tumor-promoting activities. In a breast cancer mouse model, MDSCs derived from the primary tumor area were shown to secrete more EVs than those from bone marrow or spleen [170]. MDSC-EVs inhibited cytotoxic T cell and M1 macrophage functions, thus amplifying the actions of MDSCs in the TME. These findings have also been observed in an in vitro model of colorectal cancer [171]. Exhausted T cells isolated from lesions surgically removed from HCC patients can secrete EVs, as demonstrated by Wang et al. [172]. Exhausted T cell EVs can be taken up by non-exhausted CD8 T cells and are able to induce exhaustion and reduced IFNγ and IL-2 cytokine production. 

TAM-EVs induce colorectal cancer cells migration and invasion and provide significant plasticity of BRG1 expression, a key factor promoting the colorectal cancer metastasis. This dynamic and reciprocal cross-talk between colorectal cancer cells and TAM provides a new opportunity for the treatment of metastatic colorectal cancer. TAM-EVs facilitated CD8^+^ T cell exhaustion via the miR-21-5p/YOD1/YAP/β-catenin axis in HCC as described by Pu et al. [173]. TAM-EVs promote invasion and metastasis of esophageal cancer through the lncRNA AFAP1-AS1/miRNA-26a/ATF2 axis as shown by Mi et al. [174]. These observations provide novel insight on the roles of macrophage-derived EVs carrying lncRNAs in tumor pathogenesis [175]. Overall these findings enhance our knowledge on the complex interplay mediated by EVs in the tumor microenvironment and enable the identification of new targets with potential benefit for cancer patients.

## 4. EV-Mediated Immune Activation

TEV are able to induce immunoinhibitory signaling but also anti-tumor immunity [176,177]. TEVs carry TAAs such as MUC1 [178], HSP-70, damage-associated molecular patterns (DAMPs), which are transferred to immune cells and induce anti-tumor responses as shown in Figure 1 [44,179,180,181]. As such TEVs may be utilized as vaccine adjuvants and components of anti-tumor vaccines.

Ortiz-Bonilla CJ et al. [182] observed that priming with bladder cancer-derived TEV prevented tumor growth in mice. Pro-inflammatory factors were shown to be enriched in TEV using cytokine array analyses. The effect of these factors might have contributed to enhanced TME infiltration of immune cells in tumors primed with EVs.

Montfort et al. [183] observed that EVs produced by melanoma cells expressing sphingomyelinase (nSMase)-2 promoted the expression of IL12, CXCL9 and CCL19 by DCs in vitro, with associated CD4^+^ T cell activation and reduced proportion of Tregs. Lymphoma and melanoma-derived TEVs have been found to mediate and enhance DC-based anti-tumor immunity [179,184]. Glioma-TEVs reduce the proportion of Treg cells at the tumor site, attenuating immune escape [185]. Immune cells-derived EVs can also directly carry out anti-tumor functions. CD8^+^ T cell EVs are cytotoxic and can directly kill tumor cells or deplete mesenchymal tumor stromal cells [186]. DC-EVs enriched from patients with hepatocellular carcinoma reduce Treg cells and stimulate CD8^+^ T lymphocytes at the tumor site [187].

NK-EVs derived from IL-2 dependent NK-92 cells display anti-tumor activity against solid and blood cancer cells [188]. Neuroblastoma-derived EVs promote secretion of NK-EVs with enhanced cytotoxic activity [189]. In this line, NK-derived EV miR-186 inhibited neuroblastoma growth and immune escape mechanisms [168]. 

Zhou et al. [190] showed that miR-765, a negative regulator of proteolipid protein 2 (PLP2), was downregulated in an in vivo model of human uterine corpus endometrial cancer (UCEC), thus promoting tumor progression and epithelial mesenchymal transition (EMT). On the contrary, mir-765 was overexpressed in CD45RO-CD8^+^ T cells and their EVs. Treatment with these EVs reduced tumor growth via regulation of the miR-765/PLP2 axis. These observations open up new directions for the development and implementation of adjuvant therapies based on patient-derived EVs aimed at preventing cancer progression and also support the potential application of TEV in personalized medicine.

While largely displaying immunosuppressive functions in mouse cancer models [191], TAM have also been shown to display immunostimulatory activities in cancer. Cianciaruso et al. [192] observed that TAM-EVs enriched from bladder cancer tumors displayed immunostimulatory properties at both molecular and biological levels. The authors detected positive regulators of the immune response such as pattern recognition receptors (e.g., STING) and several proteins involved in TLR signaling. Analyses of the content of TAM-EVs revealed a signature associated with immunostimulatory M1-like profiles of TAM. The cargo included members of the DOCK (“dedicator of cytokinesis”) family, factors involved in intracellular signaling networks, chemotaxis and immunity, mediating the activation and recruitment of lymphocytes. Furthermore, TAM-EVs promoted proliferation of T cells and IFNγ production ex vivo, whilst EVs enriched from TAM-depleted tumors were not able to alter T cell functions. 

Further studies are required to elucidate the several mechanisms involved in TEV-mediated stimulation vs. suppression of immune cells. The TME in which TEVs exert their functions may provide the context for their immunosuppressive vs. anti-tumor activity. 

## 5. Translational Applications: Targeting EV-Mediated Tumor Immune Suppression

EVs have been exploited as therapeutic tools and delivery vehicles since the pioneering observation by Zitovgel et al. [193] that DC-derived EVs pulsed with TAAs induced CD8^+^ T cell responses and promoted the eradication of established tumors in mice. 

EVs have great potential for drug development and as therapeutic delivery systems due to properties such as biocompatibility and stability [194]. The expression of CD47 enables EVs to avoid immune rejection via ‘don’t eat me’ signaling, contributing to the prolonged circulation time of EVs in comparison to cell-based or free drug therapies. In addition, EVs are better suited to long-term storage with limited loss of function. Importantly, ligands expressed on the surface of EVs by engaging cell receptors can activate signaling pathways in both tumor and TME compartments [195].

Adhesion molecules such as CD44, CD54 and integrins, facilitate the interaction between EVs and recipient cells (e.g., immune cells, tumor cells). Preferential uptake of TEVs by a specific cell type may be driven by the expression of specific adhesion molecules [43]. For example, integrins α6β4 and αvβ5 expressed by breast and pancreatic TEVs determined their exclusive uptake by lung fibroblasts or liver macrophages, contributing to metastasis formation [8]. Targeting these specific interactions may represent a new efficacious approach.

TEVs can also be engineered to express factors for preferential uptake by immune cells and associated with immunostimulatory activity. For example, Khani et al. [196] demonstrated DC-mediated suppression of breast cancer by engineering TEVs with miR-142 and Let7i. We have recently described the utility of EVs engineered with RNA as a strategy to re-activate the immune system against cancers including myeloma [197]. Although EVs carrying RNAs, including microRNAs, hold great promise as an innovative approach in nanomedicine to fight cancer, their use in clinical practice is still limited [198,199,200,201]. 

Depletion of suppressive factors such as TGF-β in TEVs has also triggered increased uptake by DCs, thereby enhancing anti-tumor immune responses. TGF-β1 was silenced using a shRNA strategy, promoting the release of TGF-β1-depleted TEVs from leukemic cells with improved activation of the immune system directed against leukemic cells [202,203].

As EVs can transfer molecules to recipient cells, ongoing translational research is focused on developing and optimizing strategies to deliver a variety of payloads with EVs for therapeutic interventions [204]. HELA-Exos, a formulation of tumor cell-specific EVs, has been recently optimized to specifically transfer the TLR3 agonist Hiltonol and the immunogenic cell death (ICD) inducer ELANE into breast cancer cells. HELA-Exos has been shown to enhance the immunogenicity of breast cancer cells and indirectly activate tumor-infiltrating DCs in both a mouse xenograft model and patient-derived tumor organoids [205]. An EV-based immune checkpoint blockade that antagonizes the interaction between CD47 and signal regulatory protein alpha (SIRPalpha) to block the “don’t eat me” signal CD47 at the tumor site has been described. EVs harboring SIRPα variants (SIRPα-exosomes) were sufficient to induce remarkably augmented phagocytosis of tumor cells by macrophages and increased CD8^+^ T cell TME infiltration [206]. EVs can also be loaded with PH20 hyaluronidase to break down high-molecular-weight hyaluronan (HA) in the TME. The resulting oligo-HA induces DC maturation via TLR4 activation and elicits a more potent anti-tumor response. Moreover, combining EV-PH20 with anti-PD-L1 antibody provides potent tumor-specific CD8^+^ T cell immune responses as well as prominent tumor growth inhibition both in syngeneic and spontaneous breast cancer models [207]. A platform designed to cross-link tumor cells with T cells and induce a strong immune response (synthetic multivalent antibodies retargeted exosome—SMART-Exosome) was able to effectively kill tumor cells both in vitro and in vivo [208]. EVs have been engineered to deliver the STING agonist cyclic GMP-AMP (iExoSTINGa). Selective targeting of the STING pathway in APCs with iExoSTINGa was associated with superior potency compared with STINGa alone in suppressing tumor growth in melanoma. Moreover, iExoSTINGa showed superior uptake of STINGa by DCs when compared to STINGa alone, hence leading to increased accumulation of activated CD8^+^ T cells and an anti-tumor immune response [209]. 

Immune cell-derived EVs have potential for cancer immunotherapy. DC-derived EVs are able to stimulate tumor-specific immune responses when loaded with TAAs (e.g., melanoma-associated antigen 3-MAGE-A3, alpha-fetoprotein-AFP) or IFN-γ [187,210,211]. The addition of hyaluronic acid (HA), 3-(diethylamino) propylamine, mono-phosphoryl lipid A and MUC1 induces uptake of macrophage-derived EVs by DCs, the release of TAAs in the endocytic compartment and subsequent improved antigen presentation and T cell activation [212]. EVs generated from NK cell membranes are enriched in FasL and TNF, making them cytotoxic to cancer [213,214]. 

TEV may abrogate beneficial effects of anti-tumor immune therapies. TEVs derived from B-cell lymphoma were shown to bind complement, hence protecting tumor cells from complement-dependent cytolysis in vivo [123]. Adoptive NK-92 cells transfer to relapsed AML patients has not provided significant therapeutic benefits, presumably because the transferred cells encountered circulating TEV carrying an immunosuppressive cargo [149]. As mentioned above, EVs, by enriching immunotherapeutic targets such as CD20 [44,123], mediate sequestration of therapeutic antibodies, thus interfering with antibody-based therapies and reducing binding of these antibodies to tumor cells with associated inhibition of cytotoxicity directed at tumor cells. “A phase I clinical trial is currently investigating depletion of circulating EVs via a proprietary hemo-purifier device (https://clinicaltrials.gov/ct2/show/NCT04453046, accessed on 15 November 2022), neutralizing their immunosuppressive and drug resistance effects”. 

Overcoming EV-mediated immune escape by blocking TEVs’ release or inhibiting their uptake may represent an additional therapeutic approach in cancer. For example, heparin has been shown to inhibit cellular uptake of TEVs with subsequent reduction of tumor cell migration, adhesion and inhibition of tumor growth and metastasis in several cancer models [215,216,217]. Further evaluation of heparin-based TEV-targeting may provide new opportunities for inhibiting immune escape.

## 6. Limitations and Future Perspectives

As discussed elsewhere [6,197], “a lack of standardised methodologies (e.g., EV purification, quantification, labelling) and data reporting limits inter-study comparisons and the translation of EVs from bench to bedside”. This may explain the discrepancy between the large amount of published research on the role of EVs in the tumor immune microenvironment and the few applications in clinical trial settings. Ongoing research aims to develop and optimize methodologies for isolation and purification of EVs and EV-subtypes from biological samples and for accurate quantification and content analysis. Our group has demonstrated the critical importance of samples collection and preparation for EV analyses and data interpretation [6,18,197,218]. Unpublished data inform the utility of critical evaluation of pre-analytical factors for EV-RNA isolation and preparation. “The amount and type of starting material, collection tube types, protocols for RNA isolation/preparation, representing some of the critical factors to take into consideration when working and reporting on EVs” [6].

Growing knowledge suggests dissimilar functions of factors associated with EVs vs. their soluble (non-vesicular) counterparts [44]. “EV-associated Hsp70, for example, can contribute to radiotherapy resistance in tumors [219], while immunization with non-vesicular Hsp70 is associated with more positive effects [220]”. Hence functions may not be correctly attributed to EVs or soluble factors. In fact, bio-physical properties of EVs impose challenges for their isolation/purification. Furthermore, other (co-)factors often co-isolate negatively affecting the enrichment of highly purified EVs and downstream applications (e.g., proteomics, genomics—‘omics’) and data interpretation [6,218]. Understanding the specific contribution of EVs versus other components of the tumor-associated secretome (e.g., cytokines, chemokines, growth factors) would be of critical importance in developing and implementing new promising strategies targeting single versus combined components of the secretome to improve the survival of patients with cancer [221]. As demonstrated by us and others [18,222], EVs can be isolated/purified from biofluids and cell culture supernatants [1], although “a consensus on the optimal source (i.e., plasma vs. serum) and the standardization of pre-analytical factors and reporting are still lacking”. In a recent report, we show for the first time in myeloma that the depletion of human serum albumin (a highly abundant protein in blood) from small EV preparations obtained from blood plasma of myeloma patients, improves the detection of proteins when using mass-spectrometry based proteomics [6,18]. Several methods have been developed for improved EVs’ isolation/purification from different sources and for surface and internal cargo modifications [6,18,223,224,225], with position statements regularly published to provide scientists with protocols for EV isolation/purification, analyses, accurate reporting of pre-analytical variables and methods [6,197,226]. As described in this review, circulating EVs play a role as diagnostic markers and as predictive markers of therapeutic response to immunotherapy. As recently summarized by Yu et al. [227], characteristics of EVs such as their stability and their mirroring of their parental cell in terms of composition, plus the technical capability to extract low levels of signal from background noise, makes them an intriguing proposition for use as liquid biomarkers (i.e., liquid biopsy) [218,227]. However, current methods for enrichment of EVs from complex biofluids (e.g., plasma/serum) are not able to define the cell or tissue of origin, although demonstrating the source of circulating factors (e.g., EVs, cell-free RNA and DNA) is of critical importance. “Protocols tailored to minimize the activation and release of platelet-derived EVs (the most abundant EVs in blood) have been optimized for platelet-free plasma preparation. These protocols should be widely adopted and accurately reported as discussed elsewhere” [6]. The expression levels of the TAA on EVs can be inconsistent on EVs posing additional challenges in detection, purification, analyses and reproducibility. Whiteside et al. [228] have developed a method to separate tumor from non-tumor derived EVs in blood to investigate their composition and potential application as cancer biomarkers. Of note, the study of non-tumor plasma EV fractions of each patient provided insight into changes in EV composition occurring during tumor development and progression at systemic level. Prote/gen-omic strategies may provide important insights into specific cargo enrichment and the source of EVs. A recent study by Lyden et al. [7] provided a comprehensive analysis of blood-derived EVs enriched for unique proteomic signatures in solid tumors, confirming that protein packaging reflects cancer biology and is heterogeneous across 16 types of cancer. The ‘gold standard’ ultracentrifugation was utilized for EV enrichment, although known to require high starting volumes, specialized equipment and long processing time. Furthermore, ultracentrifugation determines the co-isolation of molecules that may affect downstream applications/analyses and data interpretation, making this method unsuitable in clinical settings.

“Although not demonstrating major therapeutic benefits, trials utilizing EVs have indicated that EV-based therapy is well tolerated and clinically accomplishable” [229]. The lack of standardized EV enrichment/isolation techniques, storage methods, appropriate quality controls and inter-study comparisons, amongst other factors, has hindered further translation of EVs into clinical settings. As described in this review, EVs have been engineered for targeted drug delivery and improved efficacy of anti-cancer drugs. Engineering strategies would benefit from improved isolation methods of sub-populations of EVs and a better understanding of EV biodistribution in both pre-clinical and clinical settings [229]. Furthermore, “exploitation of allogeneic EV sources for development of off-the-shelf products would provide valuable (scalable) resource for immune-therapy applications” [230]. Finally, although beyond the scope of this review, high precision and individualized approaches, however challenging, are of critical importance and need to be tested in well-designed clinical trials. Improved outcomes of clinical trials may be achieved by better patient stratification strategies, for example by accessing to cutting-edge technologies and applying growing knowledge based on the use of fully annotated registries [https://www.mrdr.net.au/, accessed on 15 November 2022] and cytogenetic analysis or based on analyses of circulating tumor cells, circulating cell-free nucleic acids, proteins and EVs [218,231]. In this regard, we also highlight the importance of biobanks (https://www.mrdr.net.au/biobank-myeloma-1000-project/, accessed on 15 November 2022), which enable large-scale analyses for the identification of specific diseases biomarkers starting from biological or digital material with fully annotated clinical and biological data. These features are essential for improving personalized medical approaches, where effective biomarker identification is a critical step for diagnosis, prognosis and therapy prediction. The generation of biobanks for specific EV-end use would be beneficial for the progress of EV-based research.

In conclusion, a growing number of clinical trials involving EVs is ongoing, although no EV-based immunotherapies are currently approved. The biological role of TEVs is controversial with some molecules expressed on TEV displaying immune activating properties while others are immune suppressive. Further investigation of EVs’ function is warranted. In-depth analyses of the TME and TEV composition and functions will improve our understanding of EV-based immune therapies failures leading to the development of rational therapeutic application of EVs to cure cancer. 

## Figures and Tables

**Table 1:** TEV-mediated tumor immune suppression.

Cancer Type (EV-Source)	Functional Molecules	Recipient/Target Cells	Functional Effect in Recipient Cells	References
Small cell lung cancer	PD-L1	T cells	Inhibition of T cell activation	[53]
Ovarian cancer	ARG1	T cells	Suppression of T cells proliferation	[48]
Ovarian cancer	SPHK1	T cells	Promotion of T cell exhaustion	[54]
Breast cancer	TβRII	T cells	CD8+ T cell exhaustion via SMAD3 and TCF1	[55]
Melanoma	miR-3187-3p, miR-498, miR-122, miR149, miR-181a/b	T cells	Inhibition of T cell activation	[56]
Leukemia	miR-19a-3p	T cells	Suppression of T cells immune function	[57]
Melanoma and squamous cell carcinoma of head and neck	FasL	T cells	Apoptosis of CD8+ T cells and Treg expansion	[58]
Head and neck cancer	PD-L1	CD8+ T cells	Decreased CD8+ T cell activation	[59]
Glioblastoma cancer	PD-L1	T cells	Suppressed T cell activation and proliferation	[47]
Breast cancer	PD-L1	T cells	Impaired activation and cancer killing potential of T cells	[60]
Prostate and melanoma	PD-L1	CD8+ T cells	Suppressed T cell activity	[61]
Non small cell lung cancer	PD-L1	CD8+ T cells	Immunosuppressive properties	[62]
Melanoma	PD-L1	T cells	Suppressed T cell functions	[52,63]
Gastric cancer	PD-L1	T cells	Apoptosis of T cells; reduced activation of peripheral blood mononuclear cells	[64]
Hepatocellular carcinoma	14-3-3ζ	T cells	Immunosuppressive phenotype	[65]
Ovarian cancer	Ganglioside GD3	T cells	Decreased T cell activation	[66]
Ovarian cancer		T cells	Suppressed T cell functions	[67]
Melanoma		CD8+ T cells and NK cells	Enhanced apoptosis and suppressed proliferation and activation of CD8+ T cells; decreased NKG2D in NK cells	[68]
Head and neck cancer		T cells and NK cells	Enhanced apoptosis of CD8+ T cells; suppressed proliferation of CD4+ T cells; decreased NKG2D in NK cells	[46]
Melanoma cancer	miRNAs	CD4+ T cells	Enhanced apoptosis of CD4+ T cells	[69]
Non-small cell lung cancer	mutant KRAS DNA	CD4+ T cells	Conversion of naïve CD4+ T cells into Treg-like cells	[70]
Breast cancer	lncRNA SNHG16	T cells	Induction of CD73+γδ1 Tregs	[71]
Bladdre, colorectal, prostate, breast cancer	CD39, CD73	T cells	Hydrolysis of ATP and generation of adenosine	[72]
Hepatocellular carcinoma	HMGB1	B cells	Expansion of Bregs via TLR2/4-MAPK signaling pathway	[73]
Pancreatic cancer	TAAs	B cells	Inhibition of complement-dependent and antibody-dependent cell-mediated cytotoxicity	[74]
Lung cancer	miR-214	Treg	Suppression of PTEN; Treg expansion	[75]
Colorectal cancer	miR-208b	Treg	Treg expansion by targeting PDCD4, tumour growth and drug resistance	[76]
Breast cancer	lncRNA SNHG16	Vδ1 T cells	Potentiation of the TGF-β1/SMAD5 pathway to upregulate CD73 expression in Vδ1 T cells	[77]
Ovarian and head and neck squamous cancers		Treg	Increased FasL, IL-10, TGF-β1, CTLA-4, granzyme B and perforin expression and Treg-mediated stronger suppression of T cell proliferation	[78]
Nasopharyngeal carcinoma	miR-24-3p; miR-891a; miR-106a-5p; miR-20a-5p; miR-1908	Treg	Downregulated ERK/STAT1/STAT3 phosphorylation with a shift of T cells towards Treg phenotype	[79]
Leukemia	4-1BBL/CD137L	Treg	Elevated expression of effector/tumour Treg markers (CD39, CCR8, CD30, TNFR2, CCR4, TIGIT, IL21R)	[80]
Pancreatic ductal adenocarcinoma	TGF-β1	NK cells	Down-regulation of NKG2D, CD107a, TNF-α, INF-γ, CD71, CD98; impaired glucose uptake ability; attenuated NK cell cytotoxic activity	[81]
Hypoxic lung adenocarcinoma cells	miR-150-5p	NK cells	Down-regulation of CD226 and functional repression of NK cells	[82]
Pancreatic cancer	miR-212-3p	DCs	Induced immune tolerance via RFXAP	[83]
Pancreatic cancer	miR-203	DCs	Inhibition of antigen presentation via TLR4/TNF-α/IL-12	[84]
Glioblastoma multiforme	LGALS9	DCs	Inhibition of antigen recognition, processing and presentation	[85]
Oral and oropharyngeal squamous cell carcinoma	miR-17-5p, miR-21, miR-16, miR-24, miR-181a, miR-23b	DCs	Impaired differentiation and maturation of mono-DCs	[86]
Prostate cancer	PGE2	DCs	Disrupted cytokine production with inhibition of T cell activation, and increased secretion of adenosine with impaired DC functions and direct pro-tumour effects	[87]
Chronic lymphocytic leukaemia and breast cancer		DCs	Inhibited DC maturation	[88]
Melanoma	miR-155, miR-125b, miR-100, miR-146a, miR-146b, let-7e, miR-125a, and miR-99b	MDSCs	Induced immunosuppressive properties	[89]
Melanoma	HSP86	MDSCs	TLR4 and NFkB activation on MDSCs, generation of PD-L1+CD11b+Gr1+ MDSCs that suppress T cell functionality	[90]
Thymoma, mammary carcinoma, and colon carcinoma	Hsp72	MDSCs	Immunosuppressive signaling via TLR2/STAT3 axis	[76]
Renal cancer	HSP70	MDSCs	MDSC proliferation and activation via TLR2 signaling to promote tumour growth and immunosuppression	[77]
Breast cancer	miR-9, miR-181a	MDSCs	Activated JAK/STAT signaling in eMDSCs through the targeting of SOCS3 and PIAS3	[78]
Breast cancer	TGF-β1, PGE2	MDSCs	MDSC accumulation and accelerated tumour growth	[91]
Hypoxic glioma cancer cells	miR-10a, miR-21	MDSCs	Promoted MDSCs expansion and activation through miR-10a/Rora/IκBα/NF-κB and miR-21/Pten/PI3K/AKT pathways	[79]
Oral squamous cell carcinoma	miR-21	MDSCs and γδ T cells	Inhibited γδ T cell functions through MDSCs	[92]
Hypoxic pancreatic cancer cells	miR-301a	Macrophages	M2 polarization via PTEN/PI3Kgamma	[80]
Lung cancer	miR-103a	Macrophages	M2 polarization	[93]
Lung cancer	EGFR	Macrophages	Lower host innate antiviral immunity through MEKK2/IRF3 axis	[94]
Melanoma, lung and squamous skin cancers	Let-7a	Macrophages	Increased OXPHOS activity and TAM via AKT/mTOR	[95]
p53-mutant cancer cells	miR-1246	Macrophages	Promoted TAM phenotype	[96]
Melanoma	miR-125b-5p	Macrophages	Promoted survival via LIPA	[97]
Hypoxic pancreatic cancer cells	miR-301a-3p	Macrophages	Induced M2 phenotype via PTEN/PI3K signaling	[80]
Pancreatic cancer	Arachidonic acid	Macrophages	Promoted M2 phenotype	[98]
Pancreatic cancer cells undergoing ferroptosis	KRAS^G12D^ protein	Macrophages	Promoted M2 polarization through STAT3-dependent fatty acid oxidation	[99]
Pancreatic cancer	miR-155-5p	Macrophages	TAM formation via EHF/Akt/NF-kB axis	[100]
Metastatic osteosarcoma cells		Macrophages	Induced M2 polarization and impaired phagocytosis, efferocytosis, and macrophage-dependent tumour cell killing	[101]
Non-small cell lung cancer		Macrophages	Induced M2 polarization and via PD-L1/HIF1α	[102]
Colorectal cancer	miR-25-3p, miR-130b-3p, miR-425-5p	Macrophages	Induced M2 polarization through suppression of PTEN and activation of PI3K/Akt signaling and contributed to the establishment of liver metastasis	[103]
Colorectal cancer		Macrophages	Mixed M1/M2 secretion pattern	[104]
Breast cancer	gp130	Macrophages	Promoted activation of STAT3 signaling, and enhanced the levels of protumourigenic cytokines and the survival of macrophages	[105]
Liver cancer cells undergoing ER stress		Macrophages	Promoted secretion of IL-6, MCP-1, IL-10 and TNF-α in macrophages through STAT3 signaling	[106]
Breast cancer cells undergoing ER stress	miR-27a-3p	Macrophages	Increase in PD-L1 expression in macrophages	[107]
Breast cancer		Macrophages	Activation of NF-κB pathway in macrophages, and enhanced levels of IL-6, TNF-α, GCSF, and CCL2 in a TLR2-dependent manner	[108]
Gastric cancer		Macrophages	Activation of NF-κB and expression of the proinflammatory factors IL-6, TNF-α, and CCL2 in macrophages to promote tumour progression	[109]
Lung cancer	miR-21, miR-29a	Macrophages	Pro-metastatic inflammatory response by serving as ligands of TLR receptors in macrophages; NF-κB activation and increasing the secretion of IL-6 and TNF-α	[110]
Hypoxic ovarian cancer cells	miR-21-3p, miR-125b-5p, miR-181d-5p	Macrophages	Differentiation into TAM	[111]
Chronic lymphocytic leukemia (CLL)	Non coding Y RNA hY4	Monocytes	Increased PD-L1	[49]
Pancreatic cancer		Monocytes	Decreased HLA-DR expression, induced arginase and ROS	[112]
Snail-expressing head and neck squamous cell carcinoma	miR-21	Monocytes	M2 polarization	[113]
Glioblastoma stem cells		Monocytes	M2 polarization and enhanced PD-L1 expression	[114]
Gastric cancer		Monocytes	Monocyte differentiation into PD-1+ TAMs, which suppress CD8+ T cell functions	[115]
Multiple myeloma	HDGF	Monocytes	MDSCs with suppressive functions	[116]
Neuroblastoma	miR-21	Monocytes	Trigger TLR8 with increased miR-155 which transfer to tumour cells leads to drug resistance	[117]

## Data Availability

Not applicable.

## Abbreviations

**A**	
AML	acute myeloid leukaemia
APCs	antigen presenting cells
ARG1	arginase-1
**B**	
BAG6	large proline-rich protein BAG6
**C**	
CCL2	C-C motif chemokine ligand 2
CCRs	chemokine receptors
CLL	chronic lymphocytic leukemia
CRC	colorectal cancer
**D**	
DAMPs	damage-associated molecular patterns
DC	dendritic cells
**E**	
eTreg	effector Treg
EGFR	epidermal growth factor receptor
EMT	epithelial mesenchymal transition
EOC	epithelial ovarian cancer
ECM	extracellular matrix
EVs	Extracellular Vesicles
**F**	
FAS-L	tumor necrosis factor ligand superfamily, member 6
FDA	Food and Drug Administration
**G**	
LGALS9	galectin 9
GBM	glioblastoma multiforme
gp100	glycoprotein 100
**H**	
HNSCC	head and neck squamous
HDGF	hepatoma-derived growth factor
HSP70	heat shock protein 70
HA	hyaluronan
HA	hyaluronic acid
**I**	
iDC	Immature DCs
ICP	immune checkpoint proteins
ICD	immunogenic cell death
IFN-β	interferon-β
**L**	
LGALS9	galectin 9
LPS	lipopolysaccharide
lnc	long non-coding
LNs	lymph nodes
CD107a/LAMP1	lysosomal-associated membrane protein 1
**M**	
MEKK2	MEK kinase 2
MART1	melanoma-associated antigen 3-MAGE-A3
MIF	macrophage migration inhibitory factor
MUC1	mucin-1
MM	multiple myeloma
MDSCs	Myeloid-derived suppressor cells
**N**	
NK	natural killer
NKG2DL	NKG2D ligand
NPM1	nucleophosmin
**P**	
PDAC	pancreatic ductal adenocarcinoma
PDCD4	programmed cell death factor 4
PD1	programmed death 1
PD-L1	programmed death ligand 1
PGE2	prostaglandin E2
PLP2	proteolipid protein 2
**R**	
RFXAP	regulatory factor X-associated protein
Tregs	regulatory T cells
**S**	
SIRPalpha	signal regulatory protein alpha
SCLC	small cell lung cancer
sNMase	neutral sphingomyelinase
nSMase	sphingomyelinase
SPHK1	sphingosine kinase-1
SPHK1	sphingosine kinase 1
SCC	squamous cell carcinoma
**T**	
TCR	T-cell receptor
TERF1	telomeric repeat binding factor 1
TβRII	TGF-β type II receptor
TLR4	Toll-like receptor 4
TGF-β	transforming growth factor β
SMAD3	transforming growth factor-beta signaling protein 3
TAAs	tumor associated antigens
TME	Tumor Microenvironment
TNF	tumor necrosis factor
TRAIL	tumor necrosis factor (TNF)-related apoptosis-inducing ligand
TAMs	tumor-associated macrophages
TEVs	tumor-derived EVs
